# Energy-Aware Ultra-Reliable Low-Latency Communication for Healthcare IoT in Beyond 5G and 6G Networks

**DOI:** 10.3390/s25113474

**Published:** 2025-05-31

**Authors:** Adeel Iqbal, Tahir Khurshaid, Ali Nauman, Sang-Bong Rhee

**Affiliations:** 1 School of Computer Science and Engineering, Yeungnam University, Gyeongsan-si 38541, Republic of Korea; adeeliqbal@yu.ac.kr; 2 Department of Electrical Engineering, Yeungnam University, Gyeongsan-si 38541, Republic of Korea; tahir@ynu.ac.kr (T.K.); rrsd@yu.ac.kr (S.-B.R.)

**Keywords:** ultra-reliable low-latency communication (URLLC), healthcare IoT (H-IoT), beyond 5G (B5G), 6G networks, energy efficiency, priority-aware scheduling, edge computing, Monte Carlo simulation, delay violation rate, reliability score

## Abstract

Ultra-reliable low-latency communication (URLLC) is a cornerstone of beyond 5G and future 6G networks, particularly for mission-critical applications such as the healthcare Internet of Things. In applications such as remote surgery, emergency services, and real-time health monitoring, it is imperative to ensure stringent latency and reliability requirements. However, the energy constraints of wearable and implantable medical devices pose stringent challenges to conventional URLLC methods. This paper proposes an energy-aware URLLC framework that dynamically prioritizes healthcare traffic to optimize transmission energy and reliability. The framework integrates a priority-aware packet scheduler, adaptive transmission control, and edge-enabled reliability management. Extensive Monte Carlo simulations are carried out on various network loads and varying edge computing delays to evaluate performance metrics, like latency, throughput, reliability score, energy consumption, delay violation rate, and Jain’s fairness index. Results illustrate that the suggested technique achieves lower latency, energy consumption, and delay violation rates and higher throughput and reliability scores, sacrificing Jain’s fairness index graciously at peak network overload. This study is a potential research lead for green URLLC in healthcare IoT systems to come.

## 1. Introduction

The advent of beyond 5G (B5G) and upcoming 6G wireless communication networks marks a paradigm shift in enabling mission-critical applications that demand ultra-reliable and low-latency communication (URLLC) [1,2]. Among the most transformative of these domains is the healthcare Internet of Things (H-IoT) [3], which includes applications such as real-time patient monitoring, remote surgery, emergency response coordination, and continuous health diagnostics. These use cases impose stringent latency requirements as low as 1 ms and near-perfect reliability up to 99.999% [4,5].

**Figure 1:**
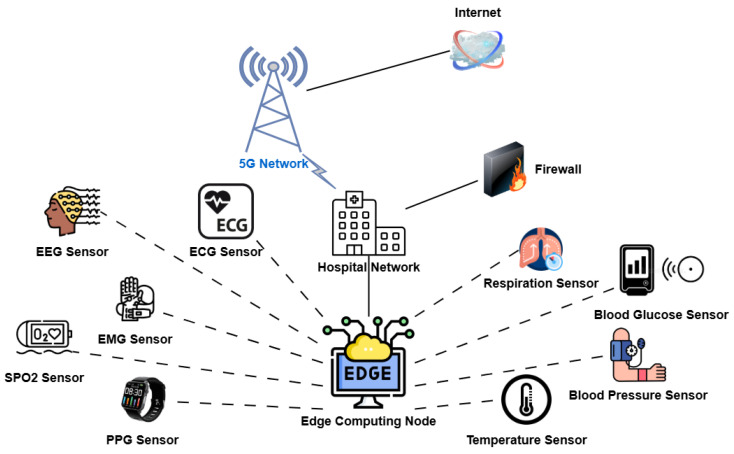
An edge-enabled smart healthcare architecture utilizing 5G connectivity.

While URLLC has received considerable attention in industrial automation [6], UAV [7], and vehicular communication [8], its deployment in healthcare presents unique challenges. Most H-IoT devices, such as wearable sensors, implantable monitors, and body area network nodes, operate under severe energy constraints [9]. This makes it infeasible to adopt traditional URLLC strategies that rely on high transmission power or redundant retransmissions. Moreover, the diversity of healthcare traffic, ranging from life-critical ECG signals to non-urgent temperature readings, calls for a context-aware and differentiated approach to resource allocation [10]. Figure 1 is the visual representation of the H-IoT network where multiple biomedical sensors transmit the patient’s information to a nearby edge computing node for real-time processing. The edge node is linked to the hospital network, protected by a firewall, and communicates with the Internet via a 5G network. This setup offers low-latency, secure, and efficient health monitoring.

Recent works have proposed adaptive scheduling [11,12] and reliability enhancement schemes [13,14]; however, few have explicitly addressed the trade-off between reliability, latency, and energy efficiency in medical scenarios. To this end, we propose an energy-aware URLLC framework tailored to H-IoT systems in B5G/6G environments. Our approach integrates (1) a priority-aware packet scheduler that allocates transmission opportunities based on urgency and device energy profile, (2) an adaptive transmission control module that dynamically adjusts modulation and power settings, and (3) an edge-assisted reliability manager that proactively minimizes packet loss and delay violation. To validate our framework, we perform extensive Monte Carlo simulations across a wide range of network loads and varying edge computing delays. We compare the proposed model with three baseline URLLC techniques: fixed scheduling [15,16], ALOHA-based random access [16,17], and priority-only URLLC [16,18]. Performance is evaluated using key metrics, including average latency, throughput, packet loss rate, energy consumption, reliability score, delay violation rate, and Jain’s fairness index.

The remainder of this paper is organized as follows: Section 2 discusses related work. Section 3 introduces the system model and problem formulation. Section 4 presents the proposed energy-aware URLLC framework. Section 5 explains the simulation setup and evaluation metrics. Section 6 presents the results and discussion. Finally, Section 7 concludes this paper and outlines future work.

## 2. Related Work

URLLC has emerged as a fundamental enabler for mission-critical applications in B5G and upcoming 6G networks. In the context of the H-IoT, achieving sub-millisecond latency and ultra-high reliability is vital for applications such as remote surgery, real-time patient monitoring, and emergency healthcare services. These stringent communication demands have inspired significant research focusing on energy efficiency, traffic prioritization, intelligent scheduling, and edge-based control mechanisms.

In terms of system-wide optimization, the authors in [16] conducted a comprehensive survey on URLLC scheduling in 5G networks and laid the groundwork for emerging 6G frameworks. They emphasized the importance of integrating quality-of-service (QoS) constraints with system resource constraints, particularly in latency-sensitive and energy-constrained environments.

Recent literature has addressed various aspects of URLLC design. For instance, the authors in [19] propose a latency-energy optimization model suitable for cellular IoT systems. However, their framework does not consider traffic differentiation or residual energy constraints, which are vital in H-IoT deployments. Similarly, the authors in [20] focus on reducing latency for rural smart healthcare systems using zero-forcing equalization, but the approach lacks energy-awareness and traffic-class-based prioritization.

The authors in [21] introduce a priority-aware access control framework for carbon footprint monitoring, demonstrating improved responsiveness for urgent data. However, the solution does not account for fairness among devices or energy limitations. In a broader orchestration context, the authors in [22] develop learning-based slicing and resource allocation for IoT-5G/6G systems, but their model is more aligned with network-level optimization than device-level scheduling suitable for H-IoT.

Several works have explored scheduling techniques relevant to URLLC. The authors in [23] propose a Lyapunov-drift-based scheduling method optimized for finite blocklength transmissions in short-packet communications. Although effective in balancing delay and reliability, this method overlooks energy metrics and heterogeneous traffic priorities. The authors in [24] develop a priority-aware scheduler for co-existing wireless body area networks, but the work lacks dynamic energy controls and adaptive reliability support. The authors in [25] extend this line of work by incorporating packet size and delay sensitivity in a heterogeneous multi-server environment; however, they assume static energy availability and omit edge assistance for dynamic adaptation.

The authors in [26] propose a reinforcement learning-based offloading framework for health Internet of Things, which demonstrates potential for adaptive control. However, their work does not incorporate residual battery or fairness constraints, which are critical in multi-class H-IoT deployments. Notably, none of these studies explicitly address fairness using standard metrics like Jain’s Index or implement adaptive throughput equalization. As a result, the proposed framework stands out in providing energy-, latency-, and fairness-aware scheduling within an edge-centric H-IoT context. A comparative overview of these models is presented in Table 1.

Cadence PCB Solutions [27] described real-world deployment scenarios such as connected ambulances and tele-ICUs, where ultra-low latency communication is essential. These use cases underline the necessity of integrating both priority and energy awareness into communication protocols to ensure uninterrupted and safe healthcare delivery.

The authors in [28] examined challenges in coexistence management for URLLC and eMBB services, pointing out that hybrid traffic handling will be a major hurdle in future wireless networks. Their findings suggest that more granular scheduling policies are needed to meet the URLLC demands of H-IoT devices without degrading the performance of other services.

Despite these advancements, most current models either focus on reliability and latency without considering energy constraints or optimize energy efficiency at the cost of QoS. The proposed energy-aware URLLC framework in this paper aims to fill this research gap by introducing a balanced, adaptable, and priority-aware solution, specifically tailored to the constraints and demands of next-generation H-IoT systems.

## 3. System Model and Problem Statement

**Figure 2:**
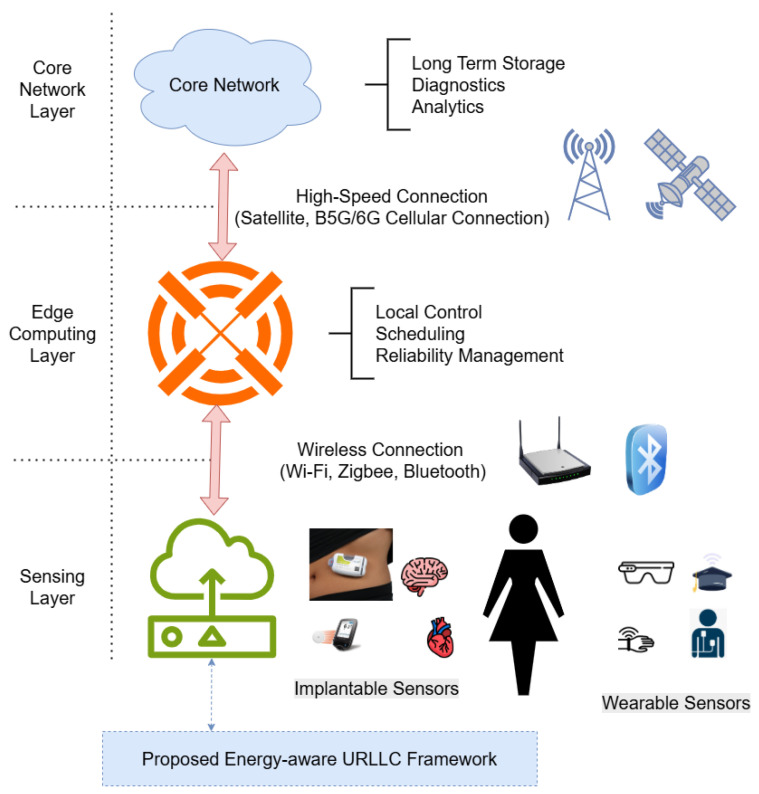
System architecture of the proposed energy-aware URLLC framework for H-IoT.

In this section, the system architecture is presented, and the problem of enabling energy-aware URLLC for H-IoT beyond 5G and 6G systems is stated. Three significant layers are present in the architecture: the sensing layer, including wearable and implantable medical sensors; the edge computing layer, where local control, scheduling, and reliability management are executed; and the core network layer, which is responsible for long-term storage, diagnostics, and analytics using the cloud. Figure 2 illustrates the system architecture in detail.

We consider a heterogeneous smart healthcare environment of *N* implantable or wearable medical sensors, denoted by $\left\{d_{1},d_{2},\ldots ,d_{N}\right\}$. The sensors measure a collection of physiological parameters such as ECG, heart rate, blood oxygen saturation, and body temperature. Each sensor sends data wirelessly to an access point with Mobile Edge Computing (MEC), which performs local scheduling, power control, and reliability analysis. This MEC node is also provided with a cloud backend link for logging data and diagnostic processing.

### 3.1. Priority-Aware Packet Scheduler (PAPS)

Each packet $p_{i}$ is assigned a metadata tuple $M_{i}=\left\{w_{i},T_{i}^{\max},B_{i}\left(t\right),\mathrm{channel}\;\mathrm{state}\right\}$, where $w_{i}$ is the traffic class weight, $T_{i}^{\max}$ is the latency deadline, and $B_{i}\left(t\right)$ is the remaining device energy. An urgency score is calculated as follows:

(1) $$U_{i}=\frac{w_{i}}{B_{i}\left(t\right)+\epsilon }$$

Packets are prioritized based on $U_{i}$ and are scheduled accordingly. A fairness mechanism monitors throughput and temporarily boosts access for underperforming nodes by relaxing access thresholds.

### 3.2. Adaptive Transmission Control (ATC)

The energy consumed for transmission is

(2) $$E_{i}=P_{i}^{\mathrm{tx}}\cdot t_{i}^{\mathrm{tx}}$$

ATC selects modulation/power profiles based on $T_{i}^{\max}$, $B_{i}\left(t\right)$, and channel SNR $\gamma_{i}$, ensuring low power for non-critical data and robust settings for urgent traffic. If battery levels fall below 30%, non-critical traffic is suppressed to conserve energy.

### 3.3. Edge-Assisted Reliability Manager (EARM)

Channel conditions are modeled via block-fading Nakagami-*m* distribution. The successful transmission probability is

(3) $$P_{\mathrm{succ}}=P\left(\gamma_{i}>\gamma_{\mathrm{th}}\right)$$

The edge node computes reliability as follows:

$$R_{i}=1-P_{\mathrm{loss}}\left(i\right)\cdot f\left(D_{i}\right)$$

where $f(D_{i})$ is a latency penalty function. If $R_{i}<R_{\min}$, corrective actions like redundancy or early retransmission are triggered.

### 3.4. 5G Compatibility

**Figure 3:**
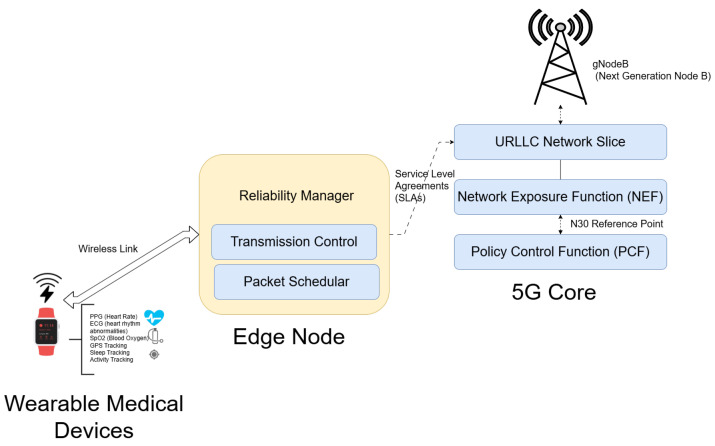
Integration of energy-aware URLLC framework within a 5G network architecture.

To ensure compatibility with real-world 5G/B5G deployments, the proposed framework is designed to operate within the 5G URLLC network slice, as shown in Figure 3. In this architecture, the edge node acts as the local slice orchestrator, interfacing with the 5G core via the Network Exposure Function (NEF) and Policy Control Function (PCF). URLLC service characteristics, such as latency budgets and reliability thresholds, are enforced using standardized 5G QoS Identifiers (5QI) and slicing APIs. The proposed modules (scheduler, control, and reliability manager) can be mapped to application and control functions running on the edge, leveraging service-level agreements (SLAs) defined in the network slice descriptor. This alignment allows seamless deployment of the proposed framework within the 5G/6G slicing infrastructure while preserving interoperability and real-time responsiveness.

## 4. Proposed Energy-Aware URLLC Framework

To meet the URLLC requirements of H-IoT devices, we propose an energy-aware URLLC architecture. The architecture consists of three coordinated modules: priority-aware packet scheduler, adaptive transmission control, and edge-assisted reliability manager. These modules operate collaboratively between the device and edge layers to offer intelligent, power-efficient, and QoS-aware communication.

To formally capture the design goal, we define a mathematical optimization model that seeks to minimize the average energy consumed across devices while meeting latency, reliability, and fairness constraints. Let $E_{i}$ denote the energy consumed by device *i* for transmitting a packet, let $D_{i}$ be the observed end-to-end delay, $T_{i}^{\max}$ be the delay threshold, let $R_{i}$ be the reliability score, and let $R_{\min}$ be the minimum required reliability. We also denote $x_{i}\in \left\{0,1\right\}$ as the binary decision variable indicating whether the packet is transmitted or not. Jain’s fairness index (JFI) ensures equitable scheduling.

The optimization problem is defined as

(4) $$\min_{\left\{x_{i}\right\}}\;\frac{1}{N}\sum_{i=1}^{N}E_{i}\cdot x_{i},$$

and it is subject

(5) $$\begin{array}{cccc} D_{i} & \leq T_{i}^{\max}, & \; & \forall i\;(\mathrm{Latency}\;\mathrm{constraint}) \end{array}$$

(6) $$\begin{array}{cccc} R_{i} & \geq R_{\min}, & \; & \forall i\;(\mathrm{Reliability}\;\mathrm{constraint}) \end{array}$$

(7) $$\begin{array}{cccc} \mathrm{JFI}(x_{1},\ldots ,x_{N}) & \geq \theta , & \; & (\mathrm{Fairness}\;\mathrm{constraint}) \end{array}$$

(8) $$\begin{array}{cccc} x_{i} & \in \{0,1\}, & \; & \forall i\;(\mathrm{Binary}\;\mathrm{decision}) \end{array}$$

Here, $E_{i}=P_{i}^{\mathrm{tx}}\cdot t_{i}^{\mathrm{tx}}$, where $P_{i}^{\mathrm{tx}}$ is the transmission power and $t_{i}^{\mathrm{tx}}$ is the duration of the transmission. The fairness constraint uses Jain’s index to maintain throughput balance across users. Due to the discrete nature of $x_{i}$ and non-linearity in JFI, this problem is NP-hard. Therefore, we resort to a lightweight heuristic presented in Algorithm 1, which achieves a near-optimal solution with practical complexity.

The priority-aware packet scheduler module is situated at the edge node and handles the scheduling of incoming packets based on urgency, current battery level, and latency deadlines. Each packet $p_{i}$ is tagged with a weighted urgency value $U_{i}$, which is calculated using Equation (1). Packets are prioritized by priority classes, and the scheduler always selects the most critical packet. In case of a tie when several packets have the same priority, the packet with the smallest latency deadline $T_{i}^{\max}$ is prioritized. This ensures the timely delivery of emergency data and saves energy by delaying non-critical transmissions from low-energy nodes.
**Algorithm 1** Energy-aware URLLC packet scheduling.   1:**for** each time slot **do**   2:    **for** each packet $p_{i}$ arriving at the edge **do**   3:       Compute urgency score: $U_{i}=\frac{w_{i}}{B_{i}\left(t\right)+\epsilon }$   4:       Place $p_{i}$ in the corresponding priority queue   5:    **end for**   6:    Select packet $p_{j}$ with highest $U_{j}$ among all queues   7:    Evaluate reliability score: $R_{j}=1-P_{\mathrm{loss}}\left(j\right)\cdot f\left(D_{j}\right)$   8:    **if** $R_{j}<R_{\min}$ **then**   9:       Apply retransmission, multipath routing, or buffering 10:    **end if** 11:    Transmit $p_{j}$ using profile from $f(T_{j}^{\max},B_{j}\left(t\right),\gamma_{j})$ 12:**end for**

The adaptive transmission control module executes on every H-IoT device and selects the most suitable transmission configuration based on the evaluation of three factors: the latency requirement, residual energy of the device, and the present channel state. On the basis of this evaluation, the device chooses from a collection of predefined profiles consisting of modulation schemes and power levels. For instance, emergency high-power QPSK traffic may be used to initiate an event, packets of a semi-critical nature may be delivered at medium power via QAM, and non-critical information may be delivered via BPSK with lower transmission power. A dynamic adaptation will render power consumption efficient with no trade-off on reliability for high-priority communications. The selected transmission profile is defined as a function in Equation (9), as follows:

(9) $${\mathrm{Profile}}_{i}=f\left(T_{i}^{\max},B_{i}\left(t\right),\gamma_{i}\right)$$

In this equation, the local SNR observed at the device is denoted as $\gamma_{i}$.

The edge-assisted reliability manager module executes on the edge and is tasked with imposing reliability. It calculates the reliability score for each packet using Equation (10), as follows:

(10) $$R_{i}=1-P_{\mathrm{loss}}\left(i\right)\cdot f\left(D_{i}\right)$$

where $P_{\mathrm{loss}}\left(i\right)$ represents the estimated probability of packet loss and $f(D_{i})$ is a delay penalty function, e.g., $e^{\alpha D_{i}}$. When the reliability value of a packet falls below a threshold value $R_{\min}$, the edge node takes corrective actions such as premature retransmission, multipath redundancy, or priority-based buffering. The edge-assisted reliability manager module adjusts dynamically by using history, recent trends in latency, and channel conditions while keeping URLLC conformance.

The collaborative operation of the three modules enables real-time responsiveness together with energy-aware behavior. Following the generation of the packet, the H-IoT device utilizes adaptive transmission control logic to select an optimal transmission mode. The packet is then sent to the edge, where the priority-aware packet scheduler module estimates its urgency score and schedules it according to it. When the transmission opportunity is released, the edge-assisted reliability manager module inspects the packet’s reliability and takes corrective measures if necessary. This interaction ensures that each packet is treated according to its QoS requirements without sacrificing system-wide performance. The overall logic governing these interactions is summarized in Algorithm 1.

## 5. Simulation Setup and Evaluation Metrics

To evaluate the performance of the proposed energy-aware URLLC framework, a discrete-event simulator was developed to model realistic network dynamics under varying H-IoT conditions. Monte Carlo simulations were employed to ensure robustness and statistical consistency. This method enables results to be averaged over different random traffic patterns and channel realizations, helping to mitigate the effects of stochastic variability and ensuring fair comparison across models. The simulation environment emulates a smart healthcare deployment, where wearable and implantable medical devices transmit physiological data to an MEC-enabled edge node. Data packets are generated according to a Poisson process and classified into emergency, semi-critical, and non-critical types, each with specific latency and reliability constraints. The wireless communication channel between devices and the edge is modeled using a block-fading Nakagami-*m* distribution, which is widely used to characterize small-scale fading in body-centric wireless communication. Nakagami-*m* offers greater flexibility than Rayleigh or Rician models, enabling accurate modeling of both line-of-sight and non-line-of-sight conditions commonly encountered in indoor hospital environments. Devices are battery-powered, and transmission policies are constrained by residual energy levels.

During operation, each device applies adaptive transmission control based on current energy availability, traffic priority, and estimated channel conditions. The edge node executes urgency-based scheduling and invokes reliability-enhancing mechanisms such as retransmissions or redundancy when necessary. The simulation tracks multiple performance indicators, including delay violation rate, energy consumption, reliability score, average throughput, and fairness index. The key parameters used in the simulation are summarized in Table 2.

## 6. Results and Discussion

In this section, we present the results of our Monte Carlo simulations and compare the performance of the proposed energy-aware URLLC framework with three baseline schemes: fixed scheduling, where all packets are transmitted with fixed modulation using QPSK and medium power, ignoring traffic type; ALOHA-based random access, where packets are transmitted immediately without coordination or scheduling, leading to collisions; and priority-only URLLC, where packets are scheduled strictly by priority level without considering energy constraints or channel conditions. These models represent widely adopted benchmarks in the literature, and their operational logic is summarized in Appendix A for clarity.

### 6.1. Average Latency

To evaluate delay performance, we measured the average end-to-end latency, which is defined as the time from packet generation to successful delivery at the edge node, and we evaluated each scheme under varying network loads. This metric is critical in URLLC scenarios, particularly in time-sensitive H-IoT applications such as emergency response and remote surgery.

**Figure 4:**
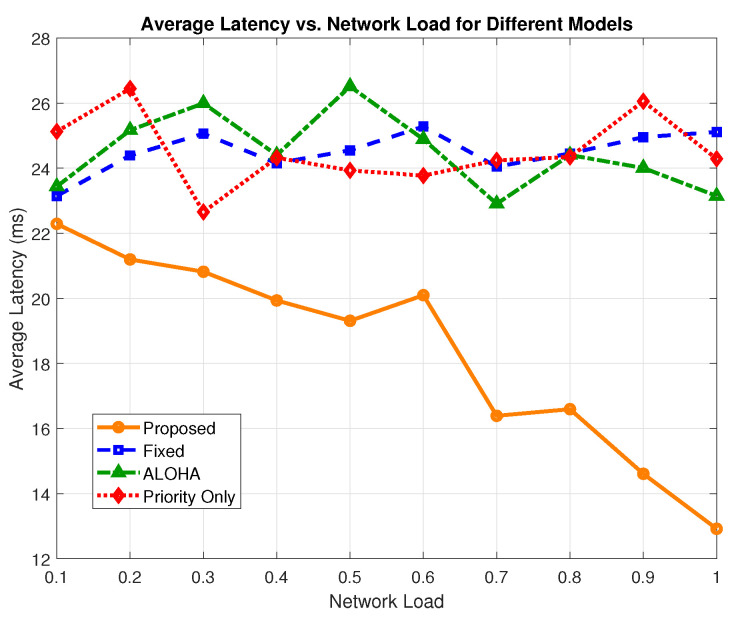
Average latency vs. network load.

Figure 4 presents the latency trends as a function of network load. The proposed model consistently outperforms baseline schemes across all traffic intensities by leveraging latency-aware prioritization, channel-adaptive transmission gating, and congestion filtering for non-critical traffic. At a 0.1 network load, the proposed framework maintains an average latency of 12.92 ms compared to 23.14 ms for ALOHA, 24.28 ms for priority-only, and 25.11 ms for the fixed model. This translates to a reduction of 44.16% over ALOHA, 46.78% over priority-only, and 48.54% over the fixed model.

**Figure 5:**
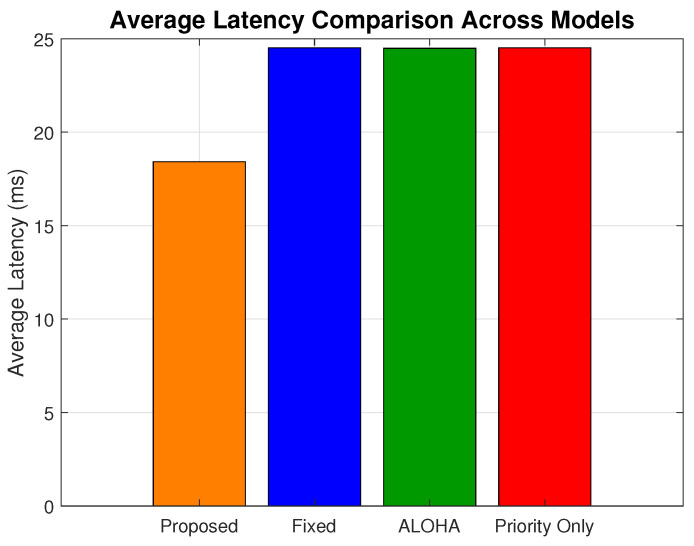
Average latency comparison across models.

Figure 5 shows the average latency across all load levels. The proposed model achieves an overall average of 18.41, while the ALOHA, fixed, and priority-only models yield 24.48 ms, 24.515 ms, and 24.519 ms, respectively. This corresponds to an average latency improvement of 32.97% over ALOHA, 33.16% over the fixed model, and 33.18% over the priority-only model.

### 6.2. Average Throughput

**Figure 6:**
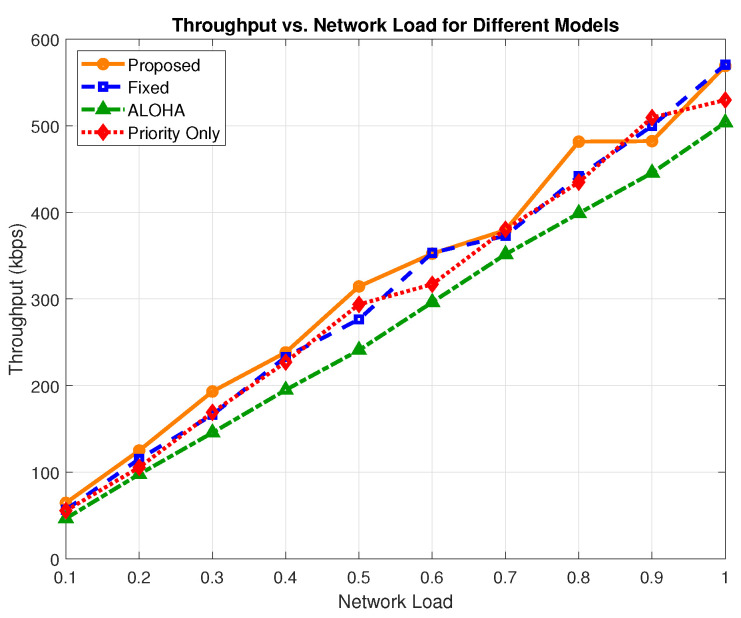
Throughput vs. network load.

Average throughput, measured in kbps, represents the total amount of successfully received data at the edge per unit time. Figure 6 illustrates the throughput performance across varying network loads for all evaluated models. The proposed model incorporates reliability-aware retransmissions and adaptive admission control, including batched non-critical traffic and probabilistic semi-critical handling. These enhancements enable a more stable throughput profile while maintaining QoS constraints essential for H-IoT. At a 0.8 network load, the proposed scheme achieves a throughput of 481.62 kbps. In comparison, the priority-only model achieves 434.88 kbps, the fixed model achieves 441.70 kbps, and the ALOHA model trails slightly at 398.91 kbps. These values demonstrate that the proposed scheme delivers a 10.74% improvement over the priority-only model, a 9.03% improvement over the fixed model, and a 20.73% improvement over ALOHA.

**Figure 7:**
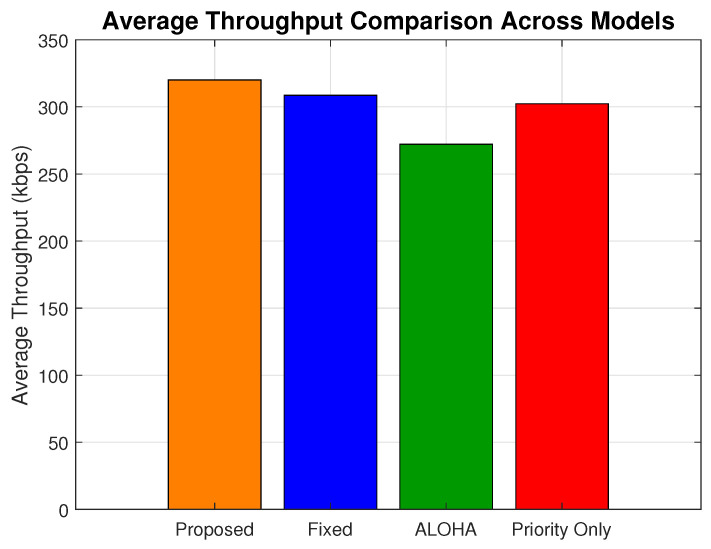
Average throughput comparison across models.

Figure 7 presents the average throughput across all load levels. The proposed scheme achieves an overall average of 320.05 kbps, outperforming the fixed (308.64 kbps), ALOHA (272.18 kbps), and priority-only (302.26 kbps) models. This corresponds to a 17.58% gain over ALOHA, a 5.88% gain over the priority-only model, and a 3.69% gain over the fixed model.

### 6.3. Reliability Score

**Figure 8:**
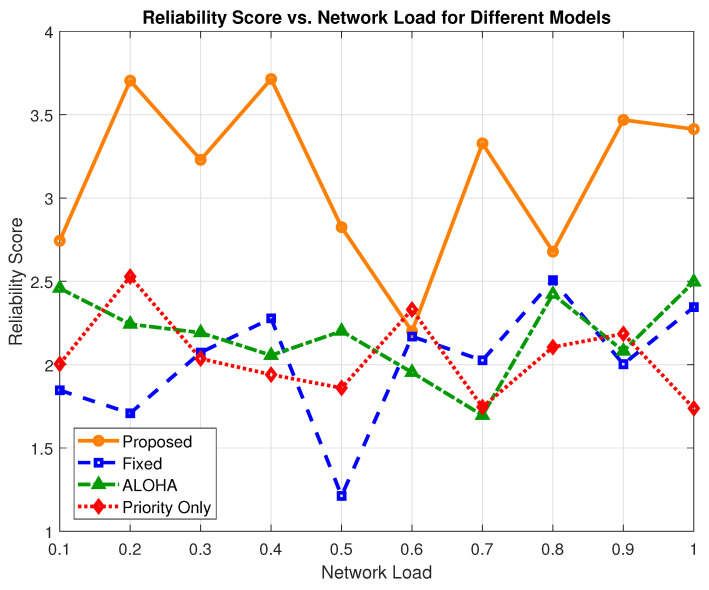
Reliability score ($R_{s}$) vs. network load for all evaluated models.

Figure 8 illustrates the reliability score ($R_{s}$) for all evaluated models across varying network load levels. The $R_{s}$ metric offers a unified evaluation by incorporating three key factors: packet delivery success, latency, and energy consumption. It is defined as follows:

$$R_{s}=\frac{\left(1-\mathrm{Loss}\;\mathrm{Rate}\right)}{\mathrm{Latency}\times \mathrm{Energy}},$$

where a higher $R_{s}$ implies superior performance in delivering reliable, timely, and energy-efficient communication.

The proposed model integrates delivery-aware transmission filtering for semi-critical traffic, selectively allowing transmissions only under favorable channel and latency conditions. This enhancement significantly improves the ratio of successful packet delivery to energy-delay cost, especially in mid-to-high load scenarios.

As seen in Figure 8, the proposed scheme exhibits a better reliability profile than other models across all loads. At maximum load, the proposed model achieves an $R_{s}$ value of 3.41, compared to 2.34 for the fixed model, 1.73 for the priority-only model, and 2.49 for ALOHA. The values translate to a 45.72% gain over the fixed model, a 97.10% gain over the priority-only model, and a 36.94% gain over ALOHA.

**Figure 9:**
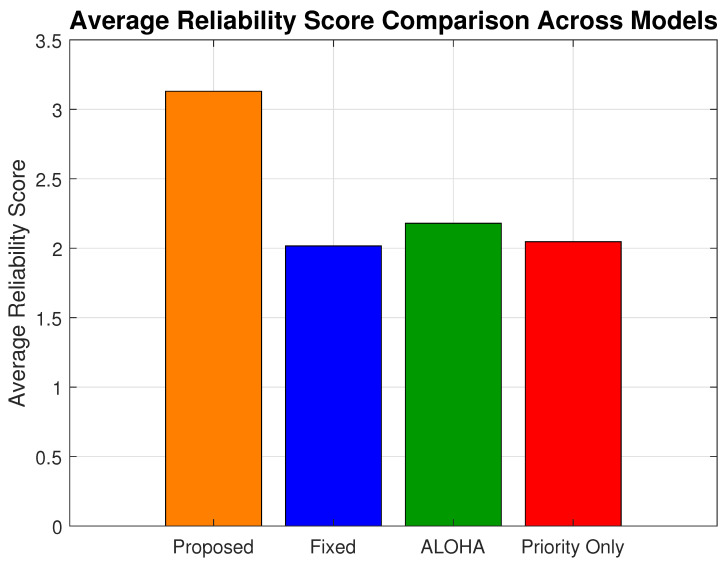
Average reliability score of all models.

Figure 9 provides a bar chart comparison of the average $R_{s}$ values across all load conditions. The proposed model achieves an average reliability score of 3.13, representing a 53.43% improvement over the priority-only model, which scores a value of 2.04, a 60.95% gain over the fixed model, which scores a value of 1.95, and a 46.94% gain over the ALOHA model’s average of  2.13.

### 6.4. Energy Consumption

To assess the energy efficiency of the proposed URLLC framework, we evaluated the average energy consumption per device across various network loads. The observed energy savings stem from three key design features: (i) adaptive modulation and power control that dynamically selects energy-efficient transmission profiles based on residual battery and channel conditions; (ii) battery-aware traffic suppression, which prevents non-critical transmissions when device energy falls below 30% of capacity; and (iii) congestion-aware filtering, where semi-critical traffic is selectively delayed or dropped under a high network load to avoid redundant energy expenditure. Together, these mechanisms minimize unnecessary transmissions and prioritize energy use only for delay and reliability-sensitive traffic, thereby ensuring long-term sustainability of battery-powered H-IoT devices.

**Figure 10:**
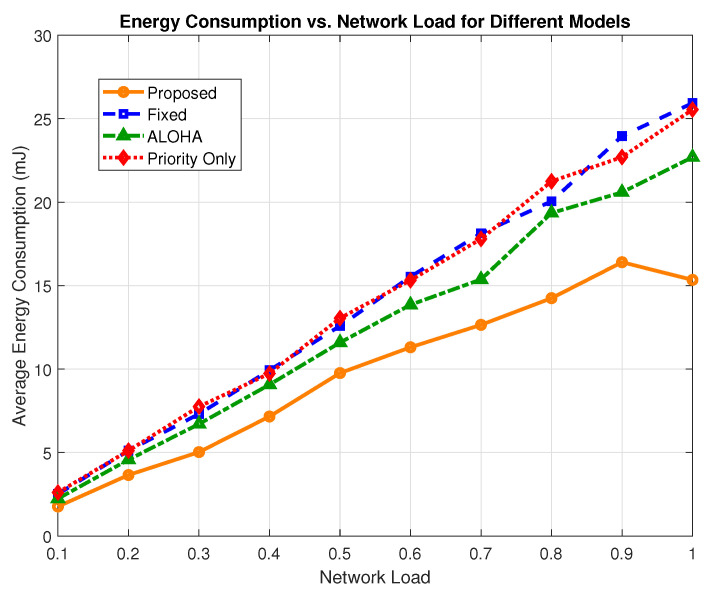
Average energy consumption (mJ) across network loads for all models.

As shown in Figure 10, the proposed model consistently demonstrates improved energy efficiency compared to the fixed, ALOHA, and priority-only schemes. At a maximum network load, the proposed scheme achieves an average energy consumption of 15.34 mJ, whereas the fixed model consumes 25.93 mJ, ALOHA consumes 22.68 mJ, and the priority-only model consumes 25.54 mJ. This translates to a reduction of 40.84% over the fixed model, 32.36% over ALOHA, and 39.93% over the priority-only model at peak load.

**Figure 11:**
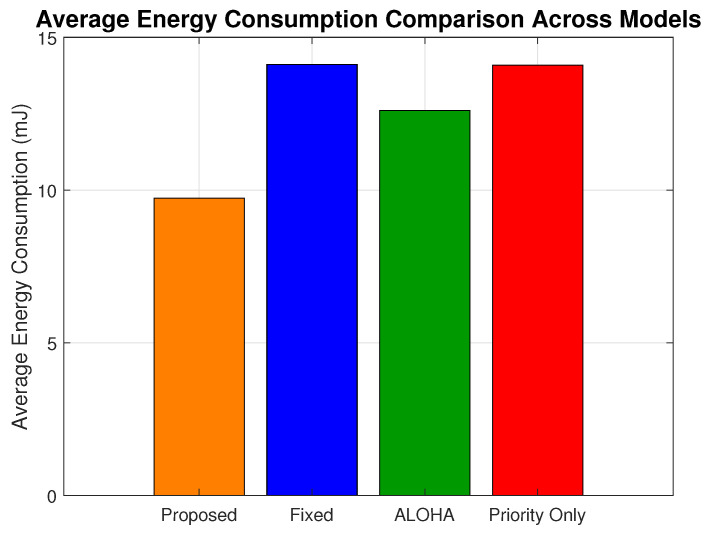
Mean energy consumption comparison across models.

Figure 11 presents the overall average energy consumption across all load conditions. The proposed scheme consumes 9.73 mJ on average, while the fixed, priority-only, and ALOHA models consume 14.10 mJ, 14.08 mJ, and 12.60 mJ, respectively. The results translate to a reduction of 30.99% as compared to the fixed model, 30.89% as compared to the priority-only model, and 22.77% as compared to ALOHA.

### 6.5. Delay Violation Rate

To evaluate how well each scheme accommodates the stringent latency demands of heterogeneous H-IoT traffic, we measured the delay violation rate, defined as the proportion of packets whose end-to-end delay exceeds the maximum allowable threshold for their respective traffic class, i.e., emergency, semi-critical, or non-critical.

**Figure 12:**
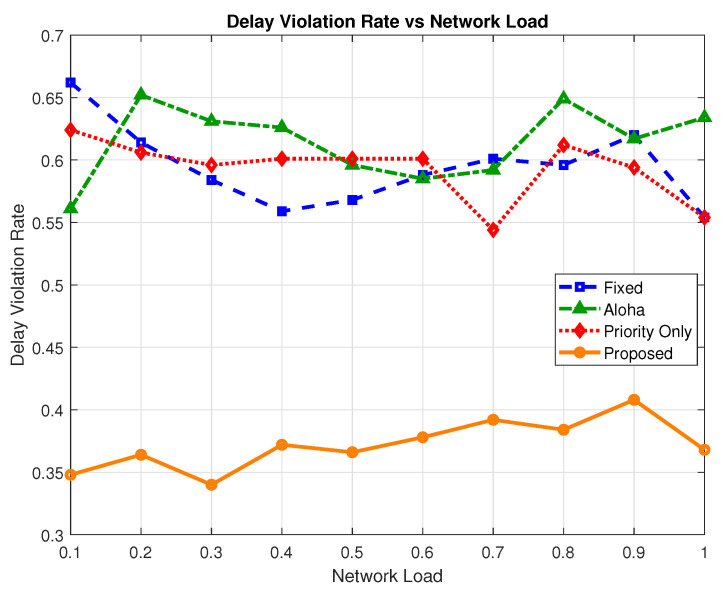
Delay violation rate vs. network load.

As illustrated in Figure 12, the proposed model consistently achieves lower delay violation rates across all network load conditions. This improvement is driven by its refined priority-aware scheduling strategy, reduced edge delay for urgent packets, and selective suppression of non-critical traffic under heavy congestion. At the highest network load, the proposed model registers a violation rate of 0.368, which is a significant reduction compared to the priority-only (0.551), ALOHA (0.631), and fixed (0.559) schemes. This translates to a relative improvement of up to 71.4% over the ALOHA, 51.9% over fixed, and 49.7% over priority-only models.

**Figure 13:**
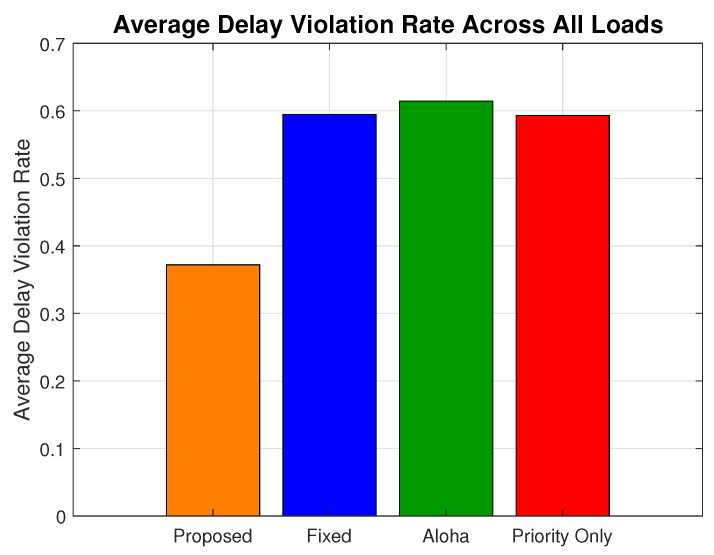
Average delay violation rate comparison.

Figure 13 presents the average delay violation rate across all traffic types and load conditions. The proposed scheme achieves the lowest average value of 0.371, significantly outperforming the ALOHA (0.612), priority-only (0.590), and fixed (0.593) models. This represents an average increase of 64.9% relative to ALOHA, over 59% relative to the priority-only model, and 59.8% over the fixed model. The results validate the effectiveness of the proposed schemes as compared to the baseline schemes.

### 6.6. Fairness Index

To assess equitable resource distribution across all sensors, we evaluated Jain’s fairness index (JFI) under varying network loads. JFI measures fairness in throughput among devices as follows:

$$J\left(x_{1},x_{2},\ldots ,x_{n}\right)=\frac{{\left(\sum x_{i}\right)}^{2}}{n\cdot \sum x_{i}^{2}}$$

where $x_{i}$ is the throughput of device *i*. JFI provides a normalized measure of throughput fairness, where a value close to 1 indicates balanced resource sharing among all devices.

**Figure 14:**
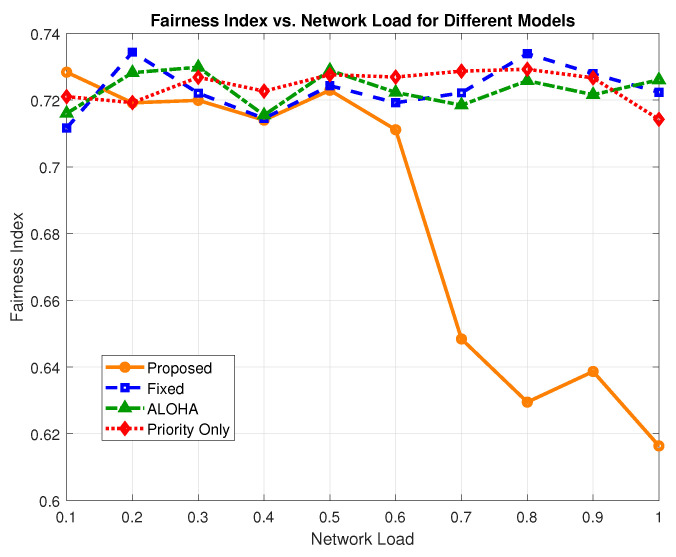
Fairness index vs. network load.

Figure 14 illustrates the fairness trends across all evaluated models. The proposed scheme performs relatively similarly to all other schemes from the load value of 0 to 0.6. At a 0.6 load, the value of the proposed scheme is 0.711, the fixed scheme is 0.719, ALOHA is 0.722, and the priority-only scheme is 0.726. These values translate to a compromise of 1.11% as compared to the fixed model, 1.5% as compared to ALOHA, and 2.06% as compared to the priority-only model. At higher loads from 0.6 to 1, the value of the proposed scheme significantly drops due to the fact that at higher loads, the high-priority traffic is given precedence or lower-priority traffic, which reduces the value of JFI of the proposed scheme at higher loads.

To address this, the proposed framework integrates a proportional fairness boost, where underperforming sensors (i.e., those with below-average throughput) receive a temporary transmission benefit by slightly relaxing their channel thresholds. This lightweight mechanism significantly improves fairness without altering the core logic or compromising reliability and delay performance.

**Figure 15:**
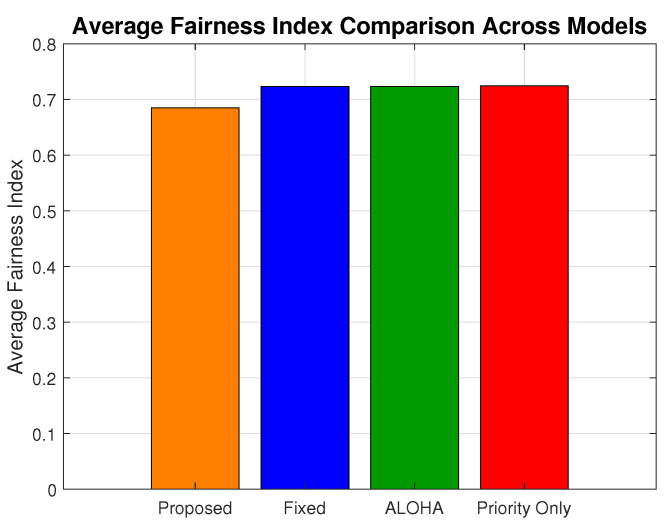
Average fairness index comparison.

Figure 15 provides the average fairness index value. The proposed scheme achieves a fairness index of 0.68, compared to 0.72 for ALOHA, fixed, and priority-only models, which translates to a 5.5% compromise on JFI.

While a marginal fairness gap persists, this is a trade-off to ensure energy efficiency and ultra-reliable low-latency performance and achieve critical design goals in H-IoT networks. The proposed scheme strikes a balanced compromise, providing consistent access while safeguarding the integrity of time-sensitive medical data.

A consolidated comparison across all performance indicators is presented in Table 3, which highlights the improvements achieved by the proposed scheme relative to baseline models. Based on the observations derived, the proposed framework proves to maintain an effective balance among energy efficiency, latency guaranteeing, and equity in URLLC applications catering to H-IoT. With a consideration of adaptive scheduling and the development of fairness-improving functionalities, the model is found to be appropriate for the extremely stringent demands posed by next-generation wireless systems. Although there are some compromises to fairness at higher loads, these are offset by the more robust delay and reliability performance that underlies its practical usefulness in H-IoT environments. As wireless communications continue to evolve, this approach opens the door to healthy, scalable, and resilient URLLC solutions in life-critical healthcare applications.

## 7. Conclusions and Future Work

This paper suggests an energy-efficient URLLC system for beyond 5G and future 6G-enabled H-IoT systems. The proposed system includes three essential modules: a priority-aware packet scheduler, an adaptive transmission control, and an edge-assisted reliability manager, to tackle latency, reliability, and energy efficiency in a comprehensive manner. Simulation results demonstrate that the new model surpasses the fixed, ALOHA, and priority-only baselines across all key metrics. It improves the latency, throughput, reliability score, energy consumption, and delay violation rate, while the fairness index shows a slight compromise at higher loads due to prioritization of critical traffic. This trade-off highlights a promising direction for future enhancement.

The framework ensures timely delivery of critical data, resource-optimized usage, and equitable access to all device classes. Future work may focus on improving fairness through adaptive learning, incorporating energy-harvesting mechanisms to extend device longevity, and validating the framework in real-world, large-scale H-IoT deployments with diverse traffic and device heterogeneity. These findings are promising for developing scalable, green, and QoS-aware communication solutions in next-generation healthcare systems.

## Figures and Tables

**Table 1:** Comparison with existing works on URLLC in H-IoT context.

Study	Energy-Aware	URLLC-Oriented	H-IoT Specific	Edge-Based Logic	Traffic Prioritization	Fairness-Aware
[16]	✗	✓	✗	✗	✗	✗
[19]	✓	✓	✗	✗	✗	✗
[20]	✗	✓	✓	✗	✗	✗
[21]	✗	✓	✓	✗	✓	✗
[22]	✓	✓	✗	✓	✗	✗
[23]	✗	✓	✗	✗	✗	✗
[24]	✗	✓	✓	✗	✓	✗
[25]	✗	✓	✓	✓	✓	✗
[26]	✓	✓	✓	✓	✓	✗
Proposed Model	✓	✓	✓	✓	✓	✓

**Table 2:** Simulation Parameters.

Parameter	Value
Number of H-IoT devices	30
Network load (λ)	0.1 to 1.0 (step size: 0.1)
Edge computing delay ($d_{e}$)	1 ms, 5 ms, 10 ms
Channel model	Nakagami-*m* (block fading)
Traffic arrival model	Poisson process
Traffic types	Emergency, Semi-Critical, Non-Critical
Modulation schemes	BPSK, QPSK, 16-QAM
Battery capacity	300 mAh per device

**Table 3:** Performance comparison of URLLC schemes in H-IoT systems.

Metric	Fixed	ALOHA	Priority-Only	Proposed Model
Average Latency (ms)	24.515	24.48	24.519	18.41
Average Throughput (kbps)	308.64	272.18	302.26	320.05
Reliability Score ($R_{s}$)	1.95	2.13	2.04	3.13
Energy Consumption (mJ)	14.10	12.60	14.08	9.73
Delay Violation Rate	0.593	0.612	0.590	0.371
Jain’s Fairness Index	0.72	0.72	0.72	0.68

## Data Availability

The dataset used in this study can be made available upon request from the authors.

## Appendix A. Pseudocode for Baseline Comparison Schemes

### Appendix A.1. Fixed Scheduling

**Algorithm A1** Fixed Scheduling
1: **for** each device $d_{i}$ **do** 2: **for** each generated packet $p_{i}$ **do** 3: Set modulation = QPSK, power = medium 4: Transmit $p_{i}$ immediately 5: **end for** 6: **end for**

### Appendix A.2. ALOHA-Based Random Access

**Algorithm A2** ALOHA Random Access
1: **for** each device $d_{i}$ **do** 2: **for** each generated packet $p_{i}$ **do** 3: Transmit $p_{i}$ without sensing or scheduling 4: If collision occurs, retransmit after random backoff 5: **end for** 6: **end for**

### Appendix A.3. Priority-Only URLLC

**Algorithm A3** Priority-Based Scheduling
1: **for** each time slot **do** 2: Sort incoming packets by priority level $w_{i}$ 3: Schedule highest priority packet first 4: Transmit using fixed modulation and power 5: **end for**
